# Vitamin D receptor gene polymorphisms and haplotypes in the etiology of recurrent miscarriages

**DOI:** 10.1038/s41598-021-84317-3

**Published:** 2021-02-25

**Authors:** Hubert Wolski, Grażyna Kurzawińska, Marcin Ożarowski, Aleksandra E. Mrozikiewicz, Krzysztof Drews, Tomasz M. Karpiński, Anna Bogacz, Agnieszka Seremak-Mrozikiewicz

**Affiliations:** 1 Division of Perinatology and Women’s Diseases, Poznan University of Medical Sciences, 60-535 Poznan, Poland; 2 Division of Obstetrics and Gynecology, Hospital Zakopane, 34-500 Zakopane, Poland; 3 Laboratory of Molecular Biology in Division of Perinatology and Women’s Diseases, Poznan University of Medical Sciences, 60-535 Poznan, Poland; 4 Department of Biotechnology, Institute of Natural Fibres and Medicinal Plants, 60-630 Poznan, Poland; 5 Division of Infertility and Reproductive Endocrinology, Poznan University of Medical Sciences, 60-535 Poznan, Poland; 6 Chair and Department of Medical Microbiology, Poznan University of Medical Sciences, 61-712 Poznan, Poland; 7 Department of Pharmacology and Phytochemistry, Institute of Natural Fibres and Medicinal Plants, 62-064 Poznan, Poland; 8 Department of Histocompatibility with Laboratory of Genetic Diagnostics, Regional Blood Center, 60-354 Poznan, Poland

**Keywords:** Molecular biology, Diseases

## Abstract

A few years ago it was shown that disturbed metabolism of the vitamin D/receptor (VD/VDR) complex may be important in the etiology of spontaneous abortion, as well as in the etiology of recurrent miscarriages (RM). The goal of this study was to investigate the association between four maternal VDR polymorphisms as well as haplotypes settings and RM occurrence in a Polish population of women in reproductive age. A total of 230 women were recruited to this study (110 with RM, 120 consecutively recruited age-matched healthy women with at least two full-term pregnancies and with no history of miscarriages). DNA samples were genotyped for *VDR* polymorphisms: *FokI* (rs2228570), *BsmI* (rs1544410), *ApaI* (rs7975232) and *TaqI* (rs731236). Significant differences in genotype distributions and allele frequencies between case and control groups were observed in *VDR BsmI* polymorphism (GG vs. GA and AA, OR = 0.56, *p* = 0.036 and OR = 1.49, *p* = 0.035, respectively). The best evidence of an association with RM prevention was observed for the TTGT haplotype, which was more frequent among controls than cases even after permutation test (0.09 vs. 0.017, *p* = 0.0024). Other haplotypes were also significantly more frequent in the control group: TGT (rs7975232, rs1544410, rs2228570), TG (rs7975232, rs1544410), TTG (rs731236, rs7975232, rs1544410), TT (rs731236, rs7975232). Our research indicated the possible role of *VDR BsmI* genetic polymorphism in RM etiology, suggesting at the same time the active role of maternal VD metabolism and its influence on pregnancy outcome. The significant influence of several maternal haplotypes was shown to prevent RM occurrence.

## Introduction

For many years the importance of the active form of vitamin D (VD—1,25-dihydroxyvitamin D3, 1,25[OH]2D3) in the processes of mineralization and bone metabolism has been known. The role of VD as a potential antiproliferative and immunomodulatory compound has also been demonstrated. Due to its potential immunomodulatory effect, vitamin D probably also affects the course and outcome of pregnancy. Recent studies indicate the association of vitamin D deficiency with adverse pregnancy outcomes, including preeclampsia, gestational diabetes, intrauterine fetal growth restriction, preterm labor and spontaneous abortions. The complex of vitamin D, vitamin D binding protein (DBP), vitamin D receptor (VDR), as well as the vitamin D activating enzymes forms an integral pathway in the human organism. The expression of these compounds is observed in many human tissues, including placenta and decidua, where they are likely to take an active part in the local autocrine and paracrine response. Additionally, attention was paid to the role of vitamin D in immunoregulation and trophoblast invasion. This suggests that local vitamin D synthesis potentially modulates the function of the placenta and the development of the fetus^1–3^. Moreover, many studies have demonstrated the role of sufficient activity of enzymes involved in VD metabolism as well as the presence of genetic polymorphism in the genes coding for enzymes and VDR in pregnant women^4–6^.

Additionally, several studies have shown increased expression of VDR and 1α-hydroxylase (CYP27B1) (enzyme that catalyzes the conversion of 25-hydroxyvitamin D3 prohormone to the active form 1,25-dihydroxyvitamin D3) in the first trimester in the trophoblast and decidua during pregnancy in humans and animals^7,8^. At the same time, VD by attaching to the VDR regulates the activity of many target genes associated with the implantation process, while the immunosuppressive effect of VD suggests its role in implant tolerance. Some studies also show abnormal expression of 1α-hydroxylase in pregnancies complicated by preeclampsia, showing the potential role of VD as a regulator of placentation^9,10^.

Recurrent miscarriages (RM), whose frequency is assessed from 1 to 3% in the population of women of reproductive age, are a serious medical, social and psychological problem. This disorder is defined as the loss of three or more pregnancies before the 24 weeks of gestation. Despite the fact that several mechanisms have been previously described for the pathogenesis of RM, the causes of about 50% of cases remain unknown^11,12^. However, for some years the multifactorial etiology of this complication, including disturbances of the immune system and vitamin D deficiency, has been known. Because of this, it appears that disturbed metabolism of VD/VDR complex by modulating the immune response may be important in the etiology of spontaneous abortion, as well as in the etiology of RM^13^. Moreover, VDR is a receptor with the pleiotropic effect of VD on human cells. Interestingly, VDR activity can be modulated by the presence of polymorphic variants in the *VDR* gene^14^. The importance of *VDR* genetic variants has been shown in several complications in pregnant women, including in preeclampsia, gestational diabetes and premature delivery^6,15–20^.

The most intensively studied *VDR* polymorphisms are *FokI* (rs2228570), *TaqI* (rs731236), *BsmI* (rs1544410), and *ApaI* (rs7975232) variants. Three single nucleotide polymorphisms (*ApaI*, *BsmI* in intron 8 section and *TaqI* in exon 9 (*Ile352Ile*) are in strong linkage disequilibrium (LD) with each other and additionally with a poly(A) length polymorphism (rs17878969) in the 3′ untranslated region of the VDR gene. These variants do not change the amino acid sequence in *VDR* protein but can influence the gene expression through regulation of mRNA stability^21^. Polymorphism *FokI* is the only known *VDR* gene variant that results in altered protein. The *FokI* variant causes a change of C>T nucleotides (ACG-ATG) in exon 2. In carriers of *C* allele (designated F) VDR protein is three amino acids shorter (424 amino acids) and more active. In contrast, individuals carrying *T* allele (designated f) synthesize a less active, full-length *VDR* protein (427amino acids)^22^.

Considering the above-mentioned observations, our study aimed highlight the association between four maternal *VDR* polymorphisms (rs2228570, rs1544410, rs7975232, rs731236) as well as *VDR* haplotype settings and RM occurrence in a Polish population of women in reproductive age.

## Results

We did not observe statistically significant differences of age, systolic and diastolic blood pressure or body mass index between studied groups. The number of miscarriages in the RM group was more than or equal to three (mean ± SD 3.45 ± 0.72). The clinical data of investigated patients are listed in Table 1.

**Table 1:** Clinical data of recurrent miscarriages (RM) women and controls.

Characteristic (mean ± SD)	RM group n = 110	Control group n = 120	*p*-value
Age (years)	32.86 ± 4.76	33.33 ± 4.14	0.43
SBP (mmHg)	116.86 ± 11.73	117.19 ± 10.45	0.82
DBP (mmHg)	73.88 ± 7.45	72.63 ± 7.83	0.22
Weight (kg)	69.55 ± 11.57	67.08 ± 10.66	0.09
Height (cm)	168.36 ± 6.23	166.91 ± 5.87	0.07
BMI (kg/m^2^)	24.55 ± 3.97	24.10 ± 3.87	0.38
Number of miscarriages	3.45 ± 0.72	0.00 ± 0.00	NA

*SBD* systolic blood pressure, *DBP* diastolic blood pressure, *BMI* body mass index.

*p*-value—T test.

The genotype distributions of the *VDR* polymorphisms were in Hardy–Weinberg equilibrium. The genotype and allele frequencies were compared between case and control subjects, and the data are shown in Table 2. Significant differences in genotype distributions and allele frequencies between case and control groups were observed in *VDR BsmI* polymorphism (GG vs. GA and AA, OR = 0.56, 95% CI 0.33–0.97, *p* = 0.036; G/A, OR = 1.49, 95% CI 1.03–2.17, *p* = 0.035, respectively).

**Table 2:** Genotype and allele frequencies of *VDR* polymorphisms among RM cases and controls.

		RM n (%)	Controls n (%)	OR (95% CI)	*p* value
**rs2228570 (FokI)**	CC	30 (27.3)	31 (25.8)	1.00 (ref)	0.332
	CT	51 (46.4)	66 (55.0)	1.25 (0.67–2.33)	
	TT	29 (26.4)	23 (19.2)	0.77 (0.37–1.61)	
Dominant	CC vs. TC-TT	80 (72.7)	89 (74.2)	1.08 (0.60–1.93)	0.805
Recessive	CC-TC vs. TT	81 (73.6)	97 (80.8)	0.66 (0.36–1.23)	0.192
Allele	C	111 (50.5)	128 (53.3)	1.00 (ref)	0.537
	T	109 (49.5)	112 (46.7)	0.89 (0.62–1.29 )	
**rs1544410 (BsmI)**	GG	33 (30.0)	52 (43.3)	1.00 (ref)	0.101
	GA	53 (48.2)	49 (40.8)	0.59 (0.33–1.05)	
	AA	24 (21.8)	19 (15.8)	0.50 (0.24–1.06)	
Dominant	GG vs. GA-AA	77 (70.0)	68 (56.7)	0.56 (0.33–0.97)	0.036
Recessive	GG-GA vs. AA	86 (78.2)	101 (84.2)	0.67 (0.35–1.31)	0.245
Allele	G	119 (54.1)	153 (63.7)	1.00 (ref)	0.035
	A	101 (45.9)	87 (36.2)	1.49 (1.03–2.17 )	
**rs7975232 (ApaI)**	GG	26 (23.6)	28 (23.3)	1.00 (ref)	0.994
	GT	59 (53.6)	64 (53.3)	1.01 (0.53–1.91)	
	TT	25 (22.7)	28 (23.3)	1.04 (0.49–2.22)	
Dominant	GG vs. TG-TT	84 (76.4)	92 (76.7)	1.02 (0.55–1.87)	0.957
Recessive	GG-GT vs. TT	85 (77.3)	92 (76.7)	1.03 (0.56–1.91)	0.913
Allele	G	111 (50.5)	120 (50.0)	1.00 (ref)	0.922
	T	109 (49.5)	120 (50.0)	0.98 (0.68–1.42 )	
**rs731236 (TaqI)**	TT	35 (31.8)	53 (44.2)	1.00 (ref)	0.145
	TC	59 (53.6)	51 (42.5)	0.57 (0.32–1.01)	
	CC	16 (14.5)	16 (13.3)	0.66 (0.29–1.49)	
Dominant	TT vs. TC-CC	75 (68.2)	67 (55.8)	0.59 (0.34–1.01)	0.054
Recessive	TT-TC vs. CC	94 (85.5)	104 (86.7)	0.90 (0.43–1.91)	0.791
Allele	T	129 (58.6)	157 (65.4)	1.00 (ref)	0.134
	C	91 (41.4)	83 (34.6)	0.75 (0.51–1.09 )	

*OR* odds ratio, *CI* confidence interval.

p value chi-square test.

The other *VDR* genotype polymorphisms were not significantly associated with RM. The allele frequencies of *VDR FokI* (C/T, *p* = 0.537), *ApaI* (G/T, *p* = 0.922) and *TaqI* (T/C, *p* = 0.134) in RM cases were similar to those in healthy controls. However, none of the results of single-marker association analysis were statistically significant after Bonferroni correction (*p* > 0.01). The calculation of the power for detecting an association between *VDR* variants and RPL was performed. Based on Hong and Marren study^23^, three or more unexplained RM prevalence 1% on average in population was assumed. The parameters used were number of cases and control, disease prevalence, significance level = 0.05, MAF for RPL cases and controls for the four tested SNPs and estimated genotypic relative risk. Assuming these parameters, we had 58.95% statistical power in this study as the average power of the tested SNPs.

Table 3 presents results of linkage disequilibrium (LD) tests noted as Dʹ and r2 between each studied locus in the *VDR* gene. The distribution of haplotype frequencies between cases and controls is shown in Table 4. Eight four-marker haplotypes with a frequency of more than 1% were detected (TGGC 26.6%, TGGT 22.9%, CTAC 19.7%, CTAT 17.0%, TTGT 5.5%, TTGC 3.0%, TTAT 2.3%, TTAC 1.9%). The best evidence of an association with RM prevention was observed for the TTGT haplotype, which was more frequent among controls than cases even after the permutation test (0.09 vs. 0.017, *p* = 0.0024). The following three-and two-marker haplotypes were also significantly more frequent in the control group: TGT (rs7975232, rs1544410, rs2228570, *p* = 0.0008), TG (rs7975232, rs1544410, *p* = 0.0007), TTG (rs731236, rs7975232, rs1544410, *p* = 0.0003), TT (rs731236, rs7975232, *p* = 0.0278).

**Table 3:** Linkage disequilibrium across the study SNPs.

	rs731236	rs7975232	rs1544410	rs2228570
rs731236	–	0.960	0.949	0.058
rs7975232	0.566	–	1.000	0.027
rs1544410	0.793	0.697	–	0.025
rs2228570	0.002	0.001	0.000	–

D’ is shown above the diagonal; r^2^ is shown below the diagonal.

**Table 4:** Haplotype analysis of SNPs genotyped in *VDR* gene.

rs731236	rs7975232	rs1544410	rs2228570	Frequency (overall)	Frequency (case, control)	χ^2^	*p* value	*p value
T	G			0.495	0.503, 0.487	0.128	0.7203	1.0000
C	T			0.371	0.412, 0.332	3.146	0.0761	0.2534
T	T			0.127	0.083, 0.168	7.390	0.0066	0.0278
	G	G		0.502	0.505, 0.500	0.009	0.9224	1.0000
	T	A		0.409	0.459, 0.362	4.431	0.0353	0.1332
	T	G		0.089	0.036, 0,138	14.461	< 0.001	0.0007
		G	C	0.302	0.271, 0.331	1.999	0.1574	0.4707
		G	T	0.289	0.270, 0.306	0.723	0.395	0.8330
		A	C	0.217	0.234, 0.202	0.683	0.4084	0.8614
		A	T	0.191	0.225, 0.160	3.111	0.0778	0.2764
T	G	G		0.495	0.504, 0.487	0.132	0.7165	1.0000
C	T	A		0.367	0.409, 0.329	3.162	0.0754	0.2796
T	T	G		0.085	0.032, 0.133	15.207	< 0.001	0.0003
T	T	A		0.042	0.050, 0.034	0.791	0.3738	0.9218
	G	G	C	0.271	0.254, 0.286	0.592	0.4417	0.9461
	G	G	T	0.231	0.250, 0.214	0.858	0.3543	0.8795
	T	A	C	0.215	0.230, 0.201	0.606	0.4364	0.9425
	T	A	T	0.194	0.229, 0.162	3.273	0.0704	0.2793
	T	G	T	0.055	0.016, 0.091	12.164	< 0.001	0.0008
	T	G	C	0.034	0.020, 0.047	2.508	0.1133	0.4198
T	G	G	C	0.266	0.254, 0,278	0.341	0.559	0.9979
T	G	G	T	0.229	0,251, 0.210	1.093	0.2957	0.9724
C	T	A	C	0.197	0.211, 0.185	0.503	0.4783	0.9941
C	T	A	T	0.170	0.198, 0.144	2.339	0.1261	0.4309
T	T	G	T	0.055	0.017, 0.090	11.711	< 0.001	0.0024
T	T	G	C	0.030	0.015, 0.044	3.251	0.0714	0.2488
T	T	A	T	0.023	0.030, 0.017	0.903	0.3420	0.9826
T	T	A	C	0.019	0.021, 0.017	0.071	0.7896	1.0000

p value—chi-square test.

*p value calculated using permutation test and a total of 10,000 permutations.

## Discussion

The VD shows the great influence on pregnancy development^24^. It has been demonstrated the expression of vitamin D receptor (VDR) and 1α-hydroxylase (CYP27B1) which is enzyme involved in the local VD synthesis in the chorion and placenta. This placental system is involved in transient immunosuppression by modulating the local immune system response, leading to lack of rejection of the antigenically foreign embryo and fetus by Diaz et al.^25^ and Chan et al.^1^. On the other hand in syncytiotrophoblast VD regulates the synthesis of human chorionic gonadotropin (hCG), human placental lactogen (hPL), estradiol and progesterone, which are hormones contributing to the growth and development of the placenta^26,27^.

Additionally, VD modulates the response of the immune system by regulating the synthesis of cytokines and inhibiting the proliferation of pro-inflammatory cells. The calcitriol directly inhibits Th1 lymphocytes differentiation and as the same way the production of pro-inflammatory cytokines, including IFNγ. Additionally calcitriol promotes Th2 lymphocytes differentiation and stimulates synthesis of anti-inflammatory cytokines as IL-4, IL-5, IL-10. The disturbances in this field by VD deficiency leads to serious pregnancy complication such as preterm delivery. Many researches shown that the presence of VD deficiency may increase the risk of preterm labor. The meta-analysis made by Zhou et al. in 2017 indicated a possible relationship between the occurrence of VD deficiency and preterm labor, as well as the possible protective effect of VD supplementation on the occurrence of this pregnancy complication^28^. The study performed by McDonnell et al. indicated that the women with optimal VD concentration had a 60% lower risk of preterm labor^29^. Also many others researches shown an adverse influence of VD deficiency on preterm birth occurrence as well as others pregnancy complications^30–32^.

Results of four meta-analyzes of randomized clinical trials (RTC)^33–36^, concerning the assessment of the impact of vitamin D supplementation during pregnancy on maternal and offspring health, proved that vitamin D intake resulted in significantly higher 25 (OH) D concentration in both the mother^34–36^ and in infants compared to the control group^36^. In addition, the results of a meta-analysis of 15 randomized and quasi-randomized clinical trials^34^ showed that vitamin D supplementation during pregnancy reduced the risk of preeclampsia, although this was not found in two other meta-analyzes^33,36^. The results of two meta-analyzes^33,36^ found that birth weight was significantly greater in infants whose mothers took vitamin D. The latest results of a meta-analysis of 20 randomized clinical trials^36^ showed that vitamin D supplementation significantly reduces insulin resistance, with no effect of supplementation on other outcomes in the mother (pre-eclampsia, cesarean section) and the infant (gestational age, birth length). The results of the meta-analysis by Perez-Lopez et al.^33^ showed no effect of vitamin D supplementation for preterm birth. However, it should be emphasized that last meta-analysis in Cochrane Database^35^, which included 30 trials (7033 women), allowed to state that supplementation of vitamin D probably can reduce the risk of pre-eclampsia, gestational diabetes, low birthweight and may reduce the risk of severe postpartum haemorrhage in comparison with women who received placebo or no intervention.

The potential role of impaired vitamin D metabolism in the etiology of recurrent miscarriages has been indicated in several studies, including the placental expression and serum level of VD and VDR. Most of them suggested reductions in VD and *VDR* expression as well as enzyme activity in placenta from women with RM. A large review of 11 studies covering populations with 2 or more RM indicated a significant reduction in VD levels among this group of women. Moreover, reduced expression of the *VDR* and 1α hydroxylase (CYP27B1) in placental villi and decidua of women with RM has also been reported^13^. Yan et al.^37^ studied the expression of *VDR* in the first trimester of pregnancy (7–10 gw.) in 40 women with RM and 40 healthy pregnant women. Women with RM showed weaker expression of *VDR* mRNA in villi (46% weaker expression, p < 0.0001) and in decidua (52% weaker expression, *p* < 0.0001) compared to women in the control group. Serum VDR level was lower in the RM group compared to the control (*p* = 0.003). In addition, significantly lower levels of *VDR* expression were observed in cytotrophoblast villi and stromal cells as well as in decidual glandular epithelial and stromal cells (*p* < 0.05). The study reported lower expression of *VDR* in trophoblastic, decidua and serum villi in the RM group compared to the control, suggesting that impaired *VDR* expression in the first trimester of pregnancy may be associated with the occurrence of RM^37^.

On the other hand, studies show the effect of disorders of vitamin D metabolism on the synthesis of cytokines in the immune response while pointing to a possible relationship with the occurrence of RM. In the study of Li et al.^38^, VD levels and *VDR* expression in decidua of women with RM were analyzed (study group of 30 women with RM and control group of 30 women with elective abortion). In addition, the expression of interleukins IL-17, IL-23, transforming growth factor β (TGF-β), *VDR* and 1-α-hydroxylase (CYP27B1) in the decidua taken during the curetting procedure was analyzed. In the RM group, VD, *VDR* and TGF-β expression levels were significantly lower while IL-17 and IL-23 expression levels were significantly higher compared to the control group. These results show that VD and VDR levels in decidua are associated with local immune responses and inflammatory cytokine synthesis, and at the same time suggest that VD and VDR may play a role in RM etiology^38^.

The same observations were made by Wang et al.^39^ who analyzed the expression of the 1α-hydroxylase enzyme (CYP27B1) in the maternal–fetal space in women in the first trimester of pregnancy (20 women with 1 miscarriage, 20 women with RM and 20 women in normal pregnancy). In addition, the expression of IL-10, IFN-γ, TNF-α and IL-2 cytokines was examined. Women with RM showed a lower level of CYP27B1 mRNA expression and a lower level of CYP27B1 protein in villi and mortality compared to normal pregnancy (for mRNA: p = 0.000 in villi, *p* = 0.002 in decidua and for protein level: *p* = 0.036 in villi, *p* = 0.007 in decidua). There were no differences in the location of CYP27B1, IL-10, IFN-γ, TNF-α, and IL-2 expression between the RM group and normal pregnancy. These studies show that lower CYP27B1 expression may be associated with RM etiology and indicate the importance of local VD synthesis in the maternal–fetal space in regulating cytokine response^39^.

In contrast, Tavakoli et al.^40^ indicated the importance of VD metabolism in RM etiology. The authors studied 8 women with RM and 8 healthy control women in whom expression of *VDR*, 1α-hydroxylase (CYP27B1), and 24-hydroxylase (CYP24A1) was analyzed. The comparative expression of *VDR*, CYP27B1, and CYP24A1 genes in the endometrium of women in both groups was noted, suggesting that expression disturbances of VD metabolism do not play any role in the pathomechanism of RM^40^.

No less interesting are the studies concerning the polymorphic variants of the *VDR* gene and its role in pregnancy outcome. Moreover, in the interesting study of Workalemahu et al.^41^ conducted in 506 mother–child pairs, a pleiotropic impact of VD on fetal growth was observed. Analyzing 8 polymorphisms in 5 genes *CUBN*, *LRP2*, *VDR*, *GC*, and *CYP2R1* related to VD metabolism, an effect of rs2282679 (GC) polymorphism on the increase in birth weight in the examined group of children was found. Additionally, the relationship of the rs4667591 (LRP2) variant (*p* < 0.001) with the sex and mass of born children was noted. This study suggests a link between genetic variants in the placenta involved in VD metabolism and birth weight of newborns^41^.

There are only a few studies referring to the role of *VDR* genetic variants in RM etiology. In the large study by Barišić et al.^5^ 320 women with RM from Slovenia and Croatia were analyzed to assess the relationship of *VDR* polymorphisms (*FoIk* rs222857, *Cdx2* rs11568820, and *Taq1* rs731236) with occurrence of this condition. The possible participation of *VDR FokI* polymorphism in RM etiology was demonstrated. The authors observed higher frequency of mutated variants of *VDR FokI* polymorphism in RM etiology (for CC genotype *p* = 0.036, for C allele *p* = 0.015, additionally CC vs. TT: OR = 2.21, *p* = 0.029). Other polymorphisms were not associated with RM development^5^.

The current, scientifically discussed problem is the assessment of the influence of vitamin D receptor gene polymorphisms (*BsmI*, *ApaI*, *FokI* and *TaqI*) on preterm birth and low birth weight. The results of an earlier cohort analysis^42^ showed that the maternal *FokI* polymorphism affected both the duration of pregnancy and birth weight, which decreased with increasing number of mutant alleles (A). It was found that mothers with the AA genotype, compared to mothers with GG or GA genotypes, showed an increased risk of preterm delivery (12-fold). In addition, in this study, supplementation with vitamin D was not associated with the results of anthropometric measurements of newborns and the risk of preterm birth. The results of the meta-analysis of 11 observational studies by Barchitta et al.^42^ regarding the relationship between the *VDR* polymorphism (*FokI*—7, *BsmI*—8, *ApaI*—5, *TaqI*—7 studies) and the risk of preterm birth, showed mainly the protective effect of the BsmI polymorphism against the risk of PTB in terms of the allele (A vs G: OR = 0.74; 95% CI 0.59–0.93) and in the recessive model (AA vs. GG + AG: OR = 0.62; 95% CI 0.43–0.89). Previously, Swamy et al.^43^ carried out assessment of the effect of 38 polymorphisms of *VDR* (615 pregnant women) on several birth outcomes and showed that 8 of 38 of single nucleotide polymorphisms (SNPs including *ApaI*) have been significantly associated with birth weight in black women. On the other side, Rosenfeld et al.^44^ observed that number of women with preterm birth decreased with increasing number of A allele of maternal *BsmI* polymorphism. Moreover, in group of women with previous history of spontaneous miscarriage, the risk of preterm birth was higher when their newborns had the non-mutated allele of *BsmI* or the mutated allele of *ApaI*. Recent case–control study^45^ showed that, genotypes: *BsmI*/TT, *ApaI*/AA, *FokI*/GG, and haplotype: GAG (*TaqI*/G-*ApaI*/A-*FokI*/G) have been significantly more frequent in women with preterm birth.

In our study we noted higher frequency of the genotype containing a mutated A allele (GA + AA) of *VDR BsmI* (70.0 vs. 56.7% in RM group (*p* = 0.036), as well as mutated A allele (45.9 vs. 36.2%, p = 0.035), which suggested the role of these genetic variants in *VDR* function and consequently disturbed vitamin D metabolism. In contrast to Barisić et al.^5^ we did not observe any significance of *VDR FokI* polymorphism in RM etiology. The great value of our research is the haplotype analysis that shows the preventive effect of some three- and two-marker settings of genetic variants TGT (*p* = 0.0008), TTG (*p* = 0.0003), TG (*p* = 0.0007), TT (*p* = 0.0278), and especially four-marker TTGT haplotype (0.017 vs. 0.090 p = 0.0024) on RM occurrence. These results suggest the special role of maternal setting genetic variants of the *VDR* gene in etiology of this complication.

Certainly, our study has some limitations. First, our analysis which is a type of association study was conducted on a relatively small group of patients (230 women, including 120 controls and 110 cases). We also noted that the statistical power of the tests being only 58.95% was lower than expected (80%). It is known that the statistical power of a genetic tests depend on the number of enrolled patients, prevalence of disease in the population, frequency of alleles and genotype of investigated polymorphism, and genotype relative risk. We recognize that our study is unsatisfactory due to inadequate sample size. On the other hand, it should be noted that patients were precisely classified into both groups as an ethnically homogenous and age matched case–control pair. In addition, women were carefully selected considering their obstetric history: in the RM group (equal or more than 3 miscarriages) and in the control group (without pregnancy loss, each woman with at least 2 pregnancies ended successfully with the birth of healthy baby). Such precise selection of patients ensured the appropriate level of analyzes, but made it difficult to obtain a larger group of patients. Secondly, in our study only 4 single nucleotide variants of the *VDR* gene were analyzed. Evidently there are several other genetic polymorphisms influencing VD metabolism, but considering the pivotal role of VDR, which is the central moderator of the pleiotropic effect of VD in human cells, it is clear that the analysis of these 4 variants both in single and haplotype settings is most important and could bring a lot to explain the mechanism of RM. On the other hand, our study reports interesting haplotype analysis of 4 genetic variants of the *VDR* gene indicating the possible protective influence of some settings of variants on RM manifestation. Finally, it should acknowledge that some unmeasured variables (including socio-demographic factors, lifestyle, diet, supplement usage and vitamin D dietary intake) could also influence RM occurrence what even limit the current analysis.

Considering the complex VD system, it seems clear that there are several other genetic variants influencing VD metabolism that can affect the various pregnancy complications. Among the polymorphisms involved in the VD pathway there are variants in the genes coding for vitamin D binding protein (DBP), enzymes of VD synthesis: cytochrome P450 2R1 (CYP2R1), cytochrome P450 27B1 (CYP27B1), 7-dehydrocholesterol reductase (DHCR7), as well as retinoid X receptor (RXRA). Simultaneously there are many meta-analysis emphasize the impact of prenatal VD status on the occurrence of complications in pregnancy^46–50^. It should be assumed then certain polymorphisms in the VD pathway are able to predict circulating vitamin D concentration. This hypothesis further complicate the situation and have an additional impact on the occurrence of pregnancy complications. These variants could also demonstrated their influence on RM appearance making the problem more complicated.

In conclusion, our research indicated the possible role of *VDR BsmI* genetic polymorphism in RM etiology suggesting at the same time the active role of maternal VD metabolism and its influence on pregnancy outcome. On the other hand, the significant influence of several maternal haplotypes (TTGT, TGT, TTG, TG, TT) was shown to prevent RM appearance. These observations merit further studies and should be repeated in groups with a larger number of patients.

Additionally, considering the relationship between VD deficiency, dysregulation of the immunological system and the occurrence of pregnancy complications, it seems that VD supplementation favorably affecting the immune system could have a significant impact on the placental and fetal environment and prevent RM occurrence.

The possible role of the *VDR BsmI* genetic polymorphism in the etiology of recurrent miscarriages extends and supplements the knowledge of intrauterine programming processes, which are long-term adaptive changes taking place in the developing fetus and which are a response to the unfavorable conditions of the intrauterine environment^51^. The long-term effect of unfavorable factors is most pronounced in certain periods of embryo and fetal development (critical windows) and may have a detrimental effect and cause the development of diseases in early postnatal period, including cardiovascular diseases, nervous and endocrine systems, metabolic diseases, including osteoporosis^52,53^ and may also contribute to miscarriages. In addition, the effects of programming can be passed on to the next generations through not yet fully understood pathophysiological pathways^54^. Hence, the multifaceted links of our results may provide some solutions for the health care of pregnant women at risk of miscarriage in terms of long-term physiological consequences for the fetus and also in adult offspring. Therefore, the obtained results may be important for prenatal care in the context of the entire health and life of a woman and in the aspect of preventive medicine. Our work can help develop guidelines for future practice.

## Methods

### Study population

A total of 230 women were recruited to the study: 110 women (mean age 32.86 ± 4.76 years) who experienced 3 or more unexplained RM before the 24th gestational week and 120 healthy women (mean age 33.33 ± 4.14 years). The analysis was performed as a case–control study. In the RM group we confirmed three or more consecutive pregnancy losses of idiopathic etiology, with the same partner and with no classical risk factors. From each patient in the case group the precise obstetric history was collected comprising the number of pregnancy losses, gestational weeks at the moment of pregnancy ending, and conducted laboratory and clinical analysis elucidating the cause of each miscarriage. The women were recruited to the case group according to ESHRE criteria^55^.

The healthy subjects (mean age 33.33 ± 4.14 years) underwent a structured interview to obtain the data about clinical and family history, as well as the number of pregnancies. These patients formed the control group consisting of age-matched women with a regular menstrual cycle, normal karyotype 46 XX, at least two successful pregnancies, and no history of infertility or pregnancy loss.

The exclusion criteria for both cases and controls were as follows: age ≥ 40 years at first miscarriage, twin pregnancy, chromosomal numerical and structural abnormalities in both parents (karyotyping was performed using standard protocols), uterine and cervical anatomical abnormalities (using several imaging methods), thrombophilia (excluding protein C, protein C antithrombin, factor V (R506Q)/prothrombin G20210A polymorphism), cigarette smoking or alcohol consumption, endocrinological (excluding luteal failure, diabetes mellitus, hyperprolactinemia and thyroid disease), autoimmunological (excluding antiphospholipid antibodies, such lupus anticoagulant, anti-cardiolipin antibodies, B2-glicoprotein I, antinuclear antibodies), incompatibility in Rh blood groups, infectious diseases (toxoplasmosis, rubella, HIV, HCMV, HBV, HCV) and chronic genitourinary infection (positive culture for GBS, chlamydia, mycoplasma as well as the presence of bacterial vaginosis). Patients with a history of preeclampsia, preterm birth, or placental abruption were also excluded from the study. All women from both case and control groups formed a homogeneous Caucasians population with Polish ethnicity.

The study was conducted in the Division of Perinatology and Women’s Diseases of Poznan University of Medical Sciences in Poznan. The research was approved by the Bioethics Committee of Poznan University of Medical Sciences, Poland (210/16, 456/20), and all patients gave written informed consent to participate before enrolling in the study.

### VDR genotyping

The peripheral blood was collected in EDTA tubes, from which genomic DNA samples were isolated according to the manufacturers guidelines using the QIAamp Blood Kit (Qiagen, Germany), and later stored at minus 20 °C. DNA was quantified by measuring the absorbance at 260 nm using a NanoDrop spectrophotometer (Thermo Scientific, USA). Basic information about the tested variants and their description in accordance with the guidelines of the Human Genome Variation Society (HGVS) (http://www.hgvs.org/content/guidelines)^56^ are presented in Table 5. Genotyping was performed in the Molecular Biology Laboratory of Poznan Medical Science University using the PCR/RFLP method. DNA samples were genotyped for *VDR* polymorphisms: *FokI* (rs2228570), *BsmI* (rs1544410), *ApaI* (rs7975232) and *TaqI* (rs731236) according to the previously published protocols^57–59^. The amplification was performed in a volume of 25 μL, containing 0.2 μg genomic DNA, 1X PCR buffer, 2.5 mM MgCl_2_, 0.2 mM of each dNTP, 0.1 μM of each primer (TIB Molbiol Inc., Germany), and 0.5 U DreamTaq Green DNA polymerase (Thermo Scientific, Waltham, CA, USA) using a Bio-Rad thermal cycler (California, United States). The cycling profile consisted of initial denaturation at 95 °C for 5 min, 30 cycles consisting of denaturation at 95 °C for 30 s; 30 s annealing temperature (varied according to polymorphism), and extension was at 72 °C for 30 s. The PCR product was hydrolyzed at 37 °C with 1 µL FokI restriction enzyme (Eurx, Poland). The following genotypes were obtained**:** CC 267 bp; CT 267, 197, 70 bp and TT 197, 70 bp. For the detection of the rs1544410 variant, the 821 bp PCR product was hydrolyzed at 37 °C with 1 µL of the restriction enzyme Mva12691 (BsmI) (Thermo Scientific, USA) obtaining the following genotypes: GG 646, 175 bp; GA 821, 646 175 bp; AA 821 bp. The PCR products for the rs7975232 and rs731236 variants (length 745 bp) were hydrolyzed with 1 mL of the restriction enzyme *ApaI* at 25 °C (Eurx, Poland), resulting in the following genotypes: TT 745 bp; TG 745, 528, 217 bp and GG 528 bp, 217 bp. The same length PCR product was hydrolyzed at 65 °C with 1 mL of the restriction enzyme TaqI (Eurx, Poland) to give the following fragments: TT 494, 251 bp, TC 494, 293, 251, 201 bp and CC 293, 251, 201 bp. Digested fragments were separated by electrophoresis on 2.5% agarose gel with Midori Green Advanced DNA Stain (Nippon Genetics, Europe GmbH). Genotyping methods was summarized in Supplementary Table S1.

**Table 5:** Primary information for study of the *VDR* polymorphisms.

rs no	Location (Chr. Location)	Genomic positions refer to *VDR* reference sequence NG_008731.1	Base change (Traditional Name Variant)	MAF	*p* for HWE in case/control
rs2228570	Exon 2 (chr12:47879112)	g.30920T>C	C>T	T (0.480)	0.446/0.250
rs1544410	Intron 8 (chr12:47846052)	g.63980G>A	G>A	A (0.409)	0.754/0.202
rs7975232	Intron 8 (chr12:47845054)	g.64978G>T	G>T	T (0.498)	0.445/0.455
rs731236	Exon 9 (chr12:47844974)	g.65058T>C	T>C	C (0.378)	0.268/0.506

*Chr.* Location according to the human reference sequence (GRCh38.p12), *MAF* minor allele frequency, *HWE* Hardy–Weinberg equilibrium.

HWE p-value chi-square test.

### Statistical analysis

Statistical analyses were performed with R version 3.6.0 version^60^. Continuous data were reported as mean ± standard deviation and compared using Student’s t test. Each SNP was tested for deviation from Hardy–Weinberg equilibrium (HWE) in both patients and controls using the chi-square test. Two-tailed *p* values less than 0.05 were accepted to be statistically significant.

Genotype frequencies in the study groups were evaluated by SNPassoc^61^. The differences in genotype frequencies between cases and controls were analyzed using codominant, recessive and dominant inheritance models. Bonferroni correction was applied to account for multiple comparisons, and *p*-values < 0.01 (0.05/4 SNPs) were interpreted as statistically significant. GAS (Genetic Association Study Power Calculator) was used to perform power calculations^62^. After considering sample size (cases and controls), disease allele frequency, genotype relative risk, and significance level, the overall study power (average of four tested variants) was calculated at 58.95%.

The haplotype-based association analysis was performed using Haploview version 4.2 software^63^. Linkage disequilibrium (LD) parameters R and Dʹ and haplotype frequencies were calculated. Distribution of haplotypes was compared in case and control groups with the chi-squared test. Significant p values were corrected using the 10,000-fold permutation test.

All methods were carried out in accordance with relevant guidelines and regulations.

## Supplementary Information

Supplementary Information.

## References

[1] Chan SY, Susarla R, Canovas D, Vasilopoulou E, Ohizua O, McCabe CJ (2015). Vitamin D promotes human extravillous trophoblast invasion in vitro. Placenta.

[2] Cyprian F, Lefkou E, Varoudi K, Girardi G (2019). Immunomodulatory effects of vitamin D in pregnancy and beyond. Front. Immunol..

[3] Hou H, Zhang JY, Chen D, Deng F, Morse AN, Qiu X (2020). Altered decidual and placental catabolism of vitamin D may contribute to the aetiology of spontaneous miscarriage. Placenta.

[4] Knabl J, Vattai A, Ye Y, Jueckstock J, Hutter S, Kainer F (2017). Role of placental VDR expression and function in common late pregnancy disorders. Int. J. Mol. Sci..

[5] Barišić A, Pereza N, Hodžić A, Krpina MG, Ostojić S, Peterlin B (2019). Genetic variation in the maternal vitamin D receptor FokI gene as a risk factor for recurrent pregnancy loss. J. Matern. Fetal Neonatal. Med..

[6] Rezavand N, Tabarok S, Rahimi Z, Vaisi-Raygani A, Mohammadi E, Rahimi Z (2019). The effect of VDR gene polymorphisms and vitamin D level on blood pressure, risk of preeclampsia, gestational age, and body mass index. J. Cell Biochem..

[7] Zehnder D, Bland R, Williams MC, McNinch RW, Howie AJ, Stewart PM (2001). Extrarenal expression of 25-hydroxyvitamin d(3)-1 alpha-hydroxylase. J. Clin. Endocrinol. Metab..

[8] Ganguly A, Tamblyn JA, Finn-Sell S, Chan SY, Westwood M, Gupta J (2018). Vitamin D, the placenta and early pregnancy: Effects on trophoblast function. J. Endocrinol..

[9] Evans KN, Bulmer JN, Kilby MD, Hewison M (2004). Vitamin D and placental-decidual function. J. Soc. Gynecol. Investig..

[10] Tamblyn JA, Hewison M, Wagner CL, Bulmer JN, Kilby MD (2015). Immunological role of vitamin D at the maternal-fetal interface. J. Endocrinol..

[11] Larsen EC, Christiansen OB, Kolte AM, Macklon N (2013). New insights into mechanisms behind miscarriage. BMC Med..

[12] Kaser D (2018). The status of genetic screening in recurrent pregnancy loss. Obstet. Gynecol. Clin. N. Am..

[13] Gonçalves DR, Braga A, Braga J, Marinho A (2018). Recurrent pregnancy loss and vitamin D: A review of the literature. Am. J. Reprod. Immunol..

[14] Abouzid M, Karazniewicz-Lada M, Glowka F (2018). Genetic determinants of vitamin D-related disorders, focus on vitamin D receptor. Curr. Drug Metab..

[15] Rosenfeld T, Salem H, Altarescu G, Grisaru-Granovsky S, Tevet A, Birk R (2017). Maternal-fetal vitamin D receptor polymorphisms significantly associated with preterm birth. Arch. Gynecol. Obstet..

[16] Baczyńska-Strzecha M, Kalinka J (2016). Influence of Apa1 (rs7975232), Taq1 (rs731236) and Bsm1 (rs154410) polymorphisms of vitamin D receptor on preterm birth risk in the Polish population. Ginekol. Pol..

[17] Javorski N, Lima CAD, Silva LVC, Crovella S, de Azêvedo SJ (2018). Vitamin D receptor (VDR) polymorphisms are associated to spontaneous preterm birth and maternal aspects. Gene.

[18] Apaydın M, Beysel S, Eyerci N, Pinarli FA, Ulubay M, Kizilgul M (2019). The VDR gene FokI polymorphism is associated with gestational diabetes mellitus in Turkish women. BMC Med. Genet..

[19] Farajian-Mashhadi F, Eskandari F, Rezaei M, Eskandari F, Najafi D, Teimoori B (2020). The possible role of maternal and placental vitamin D receptor polymorphisms and haplotypes in pathogenesis of preeclampsia. Clin. Exp. Hypertens..

[20] Siqueira TW, Júnior EA, Mattar R, Daher S (2019). Assessment of polymorphism of the VDR gene and serum vitamin D values in gestational diabetes mellitus. Rev. Bras. Ginecol. Obstet..

[21] Uitterlinden AG, Fang Y, Van Meurs JB, Pols HA, Van Leeuwen JP (2004). Genetics and biology of vitamin D receptor polymorphisms. Gene.

[22] Arai H, Miyamoto K, Taketani Y, Yamamoto H, Iemori Y, Morita K, Tonai T, Nishisho T, Mori S, Takeda E (1997). A vitamin D receptor gene polymorphism in the translation initiation codon: Effect on protein activity and relation to bone mineral density in Japanese women. J. Bone Miner. Res..

[23] Li Hong Y, Marren A (2018). Recurrent pregnancy loss: A summary of international evidence-based guidelines and practice. Aust. J. Gen. Pract..

[24] Weisman Y (2003). Maternal, fetal and neonatal vitamin D and calcium metabolism during pregnancy and lactation. Vitam. Rickets.

[25] Díaz L, Sánchez I, Avila E, Halhali A, Vilchis F, Larrea F (2000). Identification of a 25-hydroxyvitamin D3 1alpha-hydroxylase gene transcription product in cultures of human syncytiotrophoblast cells. J. Clin. Endocrinol. Metab..

[26] Barrera D, Avila E, Hernández G, Halhali A, Biruete B, Larrea F, Díaz L (2007). Estradiol and progesterone synthesis in human placenta is stimulated by calcitriol. J. Steroid. Biochem. Mol. Biol..

[27] Barrera D, Avila E, Hernández G, Méndez I, González L, Halhali A, Larrea F, Morales A, Díaz L (2008). Calcitriol affects hCG gene transcription in cultured human syncytiotrophoblasts. Reprod. Biol. Endocrinol..

[28] Zhou SS, Tao YH, Huang K, Zhu BB, Tao FB (2017). Vitamin D and risk of preterm birth: Up-to-date meta-analysis of randomized controlled trials and observational studies. J. Obstet. Gynaecol. Res..

[29] McDonnell SL, Baggerly KA, Baggerly CA, Aliano JL, French CB, Baggerly LL (2017). Maternal 25(OH)D concentrations ≥40 ng/mL associated with 60% lower preterm birth risk among general obstetrical patients at an urban medical center. PLoS ONE.

[30] Taneja A, Gupta S, Kaur G, Jain NP, Kaur J, Kaur S (2020). Vitamin D: Its deficiency and effect of supplementation on maternal outcome. J. Assoc. Physicians. India..

[31] Oh C, Keats EC, Bhutta ZA (2020). Vitamin and mineral supplementation during pregnancy on maternal, birth, child health and development outcomes in low- and middle-income countries: A systematic review and meta-analysis. Nutrients.

[32] Gilani S, Janssen P (2020). Maternal vitamin D levels during pregnancy and their effects on maternal-fetal outcomes: A systematic review. J. Obstet. Gynaecol. Can..

[33] Pérez-López FR, Pasupuleti V, Mezones-Holguin E, Benites-Zapata VA, Thota P, Deshpande A, Hernandez AV (2015). Effect of vitamin D supplementation during pregnancy on maternal and neonatal outcomes: A systematic review and meta-analysis of randomized controlled trials. Fertil. Steril..

[34] Palacios C, De-Regil LM, Lombardo LK, Peña-Rosas JP (2016). Vitamin D supplementation during pregnancy: Updated meta-analysis on maternal outcomes. J. Steroid. Biochem. Mol. Biol..

[35] Palacios C, Kostiuk LK, Peña-Rosas JP (2019). Vitamin D supplementation for women during pregnancy. Cochrane Database Syst. Rev..

[36] Gallo S, McDermid JM, Al-Nimr RI, Hakeem R, Moreschi JM, Pari-Keener M, Stahnke B, Papoutsakis C, Handu D, Cheng FW (2020). Vitamin D supplementation during pregnancy: An evidence analysis center systematic review and meta-analysis. J. Acad. Nutr. Diet..

[37] Yan X, Wang L, Yan C, Zhang X, Hui L, Sheng Q (2016). Decreased expression of the vitamin D receptor in women with recurrent pregnancy loss. Arch. Biochem. Biophys..

[38] Li N, Wu HM, Hang F, Zhang YS, Li MJ (2017). Women with recurrent spontaneous abortion have decreased 25(OH) vitamin D and VDR at the fetal-maternal interface. Braz. J. Med. Biol. Res..

[39] Wang LQ, Yan XT, Yan CF, Zhang XW, Hui LY, Xue M (2016). Women with recurrent miscarriage have decreased expression of 25-hydroxyvitamin D3–1α-hydroxylase by the fetal-maternal interface. PLoS ONE.

[40] Tavakoli M, Salek-Moghaddam A, Jeddi-Tehrani M, Talebi S, Kazemi-Sefat GE, Vafaei S (2015). Comparable vitamin D3 metabolism in the endometrium of patients with recurrent spontaneous abortion and fertile controls. Mol. Reprod. Dev..

[41] Workalemahu T, Badon SE, Dishi-Galitzky M, Qiu C, Williams MA, Sorensen T (2017). Placental genetic variations in vitamin D metabolism and birthweight. Placenta.

[42] Barchitta M, Maugeri A, La Rosa MC, San Lio RM (2018). The effect of vitamin D receptor gene (VDR) polymorphisms on adverse pregnancy outcomes—Including preterm birth (PTB and small for gestational age. Nutrients.

[43] Swamy GK, Garrett ME, Miranda ML, Ashley-Koch AE (2011). Maternal vitamin D receptor genetic variation contributes to infant birthweight among black mothers. Am. J. Med. Genet. A.

[44] Rosenfeld T, Salem H, Altarescu G, Grisaru-Granovsky S, Tevet A, Birk R (2017). Maternal-fetal vitamin D receptor polymorphisms significantly associated with preterm birth. Arch. Gynecol. Obstet..

[45] Dutra LV, Affonso-Kaufman FA, Cafeo FR, Kassai MS, Barbosa CP, Santos Figueiredo FW, Suano-Souza FI, Bianco B (2019). Association between vitamin D plasma concentrations and VDR gene variants and the risk of premature birth. BMC Pregnancy Childbirth.

[46] Ribamar A, Almeida B, Soares A, Peniche B, Jesus P, Cruz SPD, Ramalho A (2020). Maternal vitamin D deficiency during pregnancy and low birth weight: A systematic review and meta-analysis. Nutr. Hosp..

[47] Aguilar-Cordero MJ, Lasserrot-Cuadrado A, Mur-Villar N, León-Ríos XA, Rivero-Blanco T, Pérez-Castillo IM (2020). Vitamin D, preeclampsia and prematurity: A systematic review and meta-analysis of observational and interventional studies. Midwifery.

[48] Tous M, Villalobos M, Iglesias L, Fernández-Barrés S, Arija V (2020). Vitamin D status during pregnancy and offspring outcomes: A systematic review and meta-analysis of observational studies. Eur. J. Clin. Nutr..

[49] Zhang H, Huang Z, Xiao L, Jiang X, Chen D, Wei Y (2017). Meta-analysis of the effect of the maternal vitamin D level on the risk of spontaneous pregnancy loss. Int. J. Gynaecol. Obstet..

[50] Triunfo S, Lanzone A, Lindqvist PG (2017). Low maternal circulating levels of vitamin D as potential determinant in the development of gestational diabetes mellitus. J. Endocrinol. Investig..

[51] Seremak-Mrozikiewicz A, Barlik M, Drews K (2014). Fetal programming as a cause of chronic diseases in adult life. Ginekol. Pol..

[52] Pieńkowski W, Wolski H, Drews K, Seremak-Mrozikiewicz A (2015). Fetal programming and the etiology of osteoporosis. Ginekol. Pol..

[53] Zhu Z, Cao F, Li X (2019). Epigenetic programming and fetal metabolic programming. Front. Endocrinol. (Lausanne).

[54] Finkielstain GP, Lui JC, Baron J (2013). Catch-up growth: Cellular and molecular mechanisms. World Rev. Nutr. Diet..

[55] European Society of Human Reproduction and Embryology. *Guideline on the Management of Recurrent Pregnancy Loss* (2017) (accessed 12 August 2020). https://www.eshre.eu/Guidelines-and-Legal/Guidelines/Recurrent-pregnancy-loss/.

[56] Dunnen JT, Dalgleish R, Maglott DR, Hart RK, Greenblatt MS, McGowan JJ (2016). HGVS recommendations for the description of sequence variants: 2016 update. Hum. Mutat..

[57] Harris SS, Eccleshall TR, Gross C, Dawson HB, Feldman D (1997). The VDR start codon polymorphism (Fok-I) and bone mineral density in premenopausal American Black and White women. J. Bone Miner. Res..

[58] Morrison NA, Qi JC, Tokita A, Kelly PJ, Crofts L, Nguyen TV (1994). Prediction of bone density from Vitamin D receptor alleles. Nature.

[59] Pani MA, Knapp M, Donner H, Braun J, Baur MP, Usadel KH, Badenhoop K (2000). Vitamin D receptor allele combinations influence genetic susceptibility to type 1 diabetes in Germans. Diabetes.

[60] R Core Team R. A language and environment for statistical computing. *R Foundation for Statistical Computing, Vienna, Austria* (2019) (accessed 12 August 2020). https://www.R-project.org/.

[61] González JR, Armengol L, Solé X, Guinó E, Mercader JM, Estivill X (2007). SNPassoc: An R package to perform whole genome association studies. Bioinformatics.

[62] Purcell S, Cherny SS, Sham PC (2003). Genetic power calculator: Design of linkage and association genetic mapping studies of complex traits. Bioinformatics.

[63] Barrett JC, Fry B, Maller J, Daly MJ (2005). Haploview: Analysis and visualization of LD and haplotype maps. Bioinformatics.

