# Supplementary material for: Vitamin D receptor gene polymorphisms and haplotypes in the etiology of recurrent miscarriages
Source: Sci Rep. 2021 Feb 25;11:4646. doi: 10.1038/s41598-021-84317-3 (PMC7907056; doi:10.1038/s41598-021-84317-3)
Supplement: Supplementary file 1 — Supplementary Information. [file 41598_2021_84317_MOESM1_ESM.docx]

Supplementary Table 1. Methods used to genotype VDR polymorphisms.

| Polymorphism | primers | Enzyme | Product |
| --- | --- | --- | --- |
| rs2228570 | 5’-AGCTGGCCCTGGCACTGACTCTGCTCT-3’  5’-ATGGAAACACCTTGCTTCTTCTCCCTC-3’  Annealing: 58^0^C | FokI  37^0^C for 16 h | C (F): 267 bp  T (f): 197 bp, 70 bp |
| rs1544410 | 5’-CAACCAAGACTACAAGTACCGCGTCAGTGA-3’  5’-AACCAGCGGGAAGAGGTCAAGGG-3’  Annealing: 60^0^C | BsmI  37^0^C for 16 h | G (b): 646 bp, 175 bp  A (B): 821 bp |
| rs7975232 | 5'-CAGAGCATGGACAGGGAGCAA-3'  5'-GCAACTCCTCATGGCTGAGGTCTC-3’  Annealing: 62^0^C | ApaI  25^0^C for 16 h | T (A): 745 bp  G (a): 528 bp, 217 bp |
| rs731236 |  | TaqI  65^0^C for 16 h | T (T): 494 bp, 251 bp  C (t): 293 bp, 251 bp, 201 bp |
